# Application of Polymeric Micelles for Drug and Gene Delivery

**DOI:** 10.3390/pharmaceutics16050646

**Published:** 2024-05-10

**Authors:** Emi Haladjova, Stanislav Rangelov

**Affiliations:** Institute of Polymers, Bulgarian Academy of Sciences, Acad. G. Bonchev St. 103-A, 1113 Sofia, Bulgaria

Polymeric micelles have been extensively studied because of their ability to transfer biologically active agents, such as drugs and nucleic acids. They are formed due to the aggregation of amphiphilic block copolymers in an aqueous solution. Thus, colloidal particles, mainly in a nanoscale composed of a hydrophobic core surrounded by a hydrophilic shell, are produced. Polymeric micelles can effectively solubilize hydrophobic drugs in their core, providing large encapsulation efficiency, high bioavailability, and controlled and targeted drug release. The micellar shell protects the hydrophobic part from biological invasion but, similar to the core, can also accommodate active substances of appropriate nature or genes and nucleic acids and to serve as a carrier. Additionally, the specific micellar structure could be easily modified, allowing for a target design of polymeric delivery systems. In this regard, the design and utilization of polymeric micelles to transport and deliver drugs and nucleic acids is an active research area witnessing continuing development.

The Special Issue *Application of Polymeric Micelles for Drug and Gene Delivery* presents some recent trends and research achievements in the application of polymeric micelles for drug and gene delivery. The research papers published in the Special Issue cover the utilization of polymeric micelles for the delivery of synthetic or natural anticancer therapeutics, as theranostic systems for the simultaneous delivery of drugs, nucleic acids, and imaging agents, as devices for delivery of antibiotics or immunosuppressants. The Special Issue additionally offers extensive reviews deeply exploring the current progress of the polymeric micelle-based delivery systems for nucleic acids, proteins and peptides.

A main obstacle to the effective clinical application of the anticancer drugs is their poor water solubility. Contribution 1 describes the development of novel block copolymer nanocarriers of the phytocannabinoid cannabidiol, designed to enhance the solubility of the drug in water while achieving high encapsulation efficiency and prolonged drug release. These systems represent polymeric micelles formed from an amphiphilic block copolymer consisting of two outer hydrophilic polyglycidol blocks and a middle hydrophobic block of poly(ε-caprolactone) bearing pendant cinnamyl moieties. The comparative assessment of the antiproliferative effect of micellar cannabidiol vs. the free drug against the acute myeloid leukemia-derived HL-60 cell line and Sezary Syndrome HUT-78 demonstrated that the newly developed systems have pronounced antitumor activity. A novel micellar form of ferrocene-containing camphor sulfonamide (DK164) based on a poly(ethylene oxide)-b-poly(α-cinnamyl-ε-caprolactone-co-ε-caprolactone)-b-poly(ethylene oxide) triblock copolymer is demonstrated by Schröder et al. (contribution 2). The developed nanosystems possess good colloidal stability, high encapsulation efficacy, and sustained release profiles of the chemotherapeutic. The micellar form of the DK164 exhibited several advantages compared to the free substance, such as higher metabolic stability, better cellular uptake, improved bioavailability, and long-term activity, maintaining nearly the same biological activity and anticancer properties of the neat drug. Poly(acrylic acid)-block-poly(n-butyl acrylate) block copolymer micelles are used for encapsulation of hydrophobic anticancer benzimidazole-hydrazone drug (contribution 3). The resulting micellar carriers also possess fluorescent properties enabling intracellular imaging and cancer treatment simultaneously. These systems are found to exhibit enhanced antiproliferative and cytotoxic effects on MDA-MB-231 cells, with long-lasting effects on microtubule organization, with apoptotic alterations and preferential localization in the perinuclear space of cancer cells.

A great advantage of polymeric micellar systems is their ability to co-deliver different therapeutics, including drugs and genes. In their work, Jin et al. (contribution 4) used polymeric micelles prepared from methoxy poly(ethylene glycol)-b-poly(D,L-lactide) to encapsulate two antitumor agents, paclitaxel and sorafenib. The authors demonstrate an improved bioavailability and biocompatibility as well as a synergistic effect of the double-loaded systems for the treatment of ovarian cancer. Pluronic micelles are effectively used to improve the safety of doxorubicin through its simultaneous encapsulation with a cardioprotective agent (resveratrol) (contribution 5). A distinctive feature of these nanosystems is loading the two drugs into different micellar compartments (resveratrol in the core and doxorubicin in the shell), allowing their different release rates. The simultaneous delivery of doxorubicin and resveratrol via the micellar system enables the cytotoxicity of doxorubicin in lymphoma cells and lowers its cardiotoxicity in cardiac cells. Contribution 6 also demonstrates the simultaneous loading of drugs in polymeric micellar core and shell. In this work, mixed polymeric micelles based on a cationic poly(2-(dimethylamino)ethyl methacrylate)-b-poly(ε-caprolactone)-b-poly(2-(dimethylamino)ethyl methacrylate) and a non-ionic poly(ethylene oxide)–b-poly(propylene oxide)–b-poly(ethylene oxide) (Pluronic F127) triblock copolymers are developed. Ciprofloxacin is solubilized via hydrophobic interactions in the micellar core, while electrostatic interactions between the polycationic blocks and the drug localize the latter in the micellar shell. These nanoscopic systems demonstrate the capability to detach pre-formed Gram-positive and Gram-negative bacterial biofilms and significantly reduce their biomass. The metabolic activity of the biofilm is strongly suppressed by the ciprofloxacin-loaded mixed micelles, indicating successful drug delivery and release. Additionally, the cytotoxicity test reveals composition-dependent cell viability, allowing the fine-tuning of their biocompatibility. The advantages of co-delivery of anticancer drug doxorubicin and focal adhesion kinase (FAK) siRNA are presented in contribution 7. The approach of the authors includes the preparation of polyplex particles based on poly(2-hydroxypropyl methacrylamide-co-methylacrylate-hydrazone-pyridoxal) copolymer and siRNA that are subsequently incorporated in methoxy polyethylene glycol (mPEG)-modified liposomes. The resulting carriers are further loaded with doxorubicin providing both excellent cancer therapeutic efficacy and inhibition of translation of FAK proteins.

Quartier et al., in contribution 8, focus on using polymeric micelles to develop aqueous formulations of poorly water-soluble drugs for delivery into the skin. The loading of three different ascomycin-derived immunosuppressants in D-α-tocopherol-polyethylene glycol-based micelles is investigated. Despite the structural and physicochemical similarities of the immunosuppressants, the loaded micelles exhibit different stability, drug loading, and release kinetics, suggesting that even small variations in the physicochemical properties could affect formulation characteristics and bioavailability and biodistribution in the case of topical drug delivery. A specific stimuli-responsive micellar system with a high potential for targeted drug delivery is presented in contribution 9. This study achieves a selective responsivity to various diols of polymeric micelles formed from polystyrene-b-poly(4-vinyl pyridine)-b-poly(ethylene oxide) terpolymer by a controllable post-polymerization quaternization of the poly(4-vinyl pyridine) block. The micelles are loaded with alizarin, used as a model drug to study the encapsulation and release induced by sensing with three geminal diols: fructose, galactose, and ascorbic acid. The results indicate that only ascorbic acid induces the efficient release of alizarin through its vicinal diol group on sp2-hybridized carbons.

The impact of polymeric micelles on modern therapeutic strategies using the delivery of biomolecules such as nucleic acids, proteins, and peptides is pointed out in the Special Issue by two excellent review papers. Contribution 10 focuses on the current state of the art of polymeric micelles as carriers for nucleic acids, highlighting the delivery challenges of nucleic acids and the approaches for improving the safety and efficacy of nucleic acids after local or systemic administration. The advances in the design of polymeric micelles over the past decade, emphasizing stimuli-responsive properties for optimized delivery, including triggered and controlled release in time and space of proteins and peptides improving therapeutic efficacy and limiting side effects, are given by contribution 11.

